# Combination of Selenium and Green Tea Improves the Efficacy of Chemoprevention in a Rat Colorectal Cancer Model by Modulating Genetic and Epigenetic Biomarkers

**DOI:** 10.1371/journal.pone.0064362

**Published:** 2013-05-23

**Authors:** Ying Hu, Graeme H. McIntosh, Richard K. Le Leu, Laura S. Nyskohus, Richard J. Woodman, Graeme P. Young

**Affiliations:** 1 Flinders Centre for Innovation in Cancer, Flinders University of South Australia, Adelaide, Australia; 2 Biostatistics, Flinders Prevention, Promotion and Primary Health Care, General Practice, Flinders University of South Australia, Adelaide, Australia; Virginia Commonwealth University, United States of America

## Abstract

Dietary supplementation of selenium and green tea holds promise in cancer prevention. In this study, we evaluated the efficacies of selenium and green tea administered individually and in combination against colorectal cancer in an azoxymethane (AOM)-induced rat colonic carcinogenesis model and determined the underlying mechanisms of the protection. Four-week old Sprague-Dawley male rats were fed with diets containing 0.5% green tea extract, 1ppm selenium as selenium-enriched milk protein, or combination of 1ppm selenium and 0.5% green tea extract. Animals received 2 AOM (15 mg/kg) treatments to induce colonic oncogenesis. Rats were killed 8 or 30 wk later after the last AOM to examine the effect of dietary intervention on aberrant crypt foci (ACF) formation or tumor development. On sacrifice, colons were examined for ACF and tumors, the mRNA levels of *SFRP5* and *Cyclin D1*, and the proteins levels of ß-catenin, COX-2, Ki-67, DNMT1 and acetyl histone H3. The combination of selenium and green tea resulted in a significant additive inhibition of large ACF formation, this effect was greater than either selenium or green tea alone, *P*<0.01; the combination also had a significant additive inhibition effect on all tumor endpoints, the effect of the combination diet on tumor incidence, multiplicity and size was greater than selenium or green tea alone, *P*<0.01. Rats fed the combination diet showed marked reduction of DNMT1 expression and induction of histone H3 acetylation, which were accompanied by restoration of *SFRP5* mRNA in normal-appearing colonic crypts. The combination diet also significantly reduced ß-catenin nuclear translocation, *Cyclin D1* expression and cell proliferation. These data show, for the first time, that combination of selenium and green tea is more effective in suppressing colorectal oncogenesis than either agent alone. The preventive effect is associated with regulation of genetic and epigenetic biomarkers implicated in colonic carcinogenesis.

## Introduction

Colorectal Cancer (CRC) is one of the leading causes of cancer death worldwide; different to other cancers, the importance of environmental exposure (especially diet) in the aetiology of CRC is highlighted by the fact that <50% of the causation can be attributed to familial factors, whereas dietary factors contribute up to 70% [1]. Specific dietary strategies might prove valuable in protecting against cancer [2]. In this regard, associations between diet and lifestyle have been established for CRC prevention, and it has been estimated that 30–50% of CRC could be potentially preventable by consuming a healthy diet [3].

In recent years, naturally occurring agents present in what we eat or drink have drawn a great deal of attention with their potential ability for cancer prevention and/or treatment because of their various health benefits and wide safety margin [2], [4]. Selenium (Se), an essential trace micronutrient, and green tea, the most common beverage consumed worldwide, have been identified as chemopreventive agents for various cancers. Epidemiological, clinical and preclinical studies suggest an inverse relationship between Se intake, green tea consumption and the risk of certain cancers [5]–[7]. Studies with animal models also have shown that Se and green tea have a wide range of preventive activity against CRC [8]–[10]. Despite promising results in preclinical settings, current clinical trial data related to Se and green tea supplementation are not convincing enough to allow a general recommendation for using Se or green tea as an effective agent for chemoprevention of cancer in humans [11]–[13]. The limitation of any single dietary agent for effective prevention might be due to lower potency of dietary agents, whereas natural compounds have shown greater activity when they are present in a mixture [14]. It might be possible to achieve additive or synergistic preventive effects by combining dietary agents that exert complementary mechanisms in their anti-carcinogenic actions [2], [15], [16]. Considerable data from animal and human studies indicate that combinations of dietary agents are more effective than a single agent [17], [18]. For instance, green tea has been shown to synergistically or additively increase the efficacy of other drugs or dietary agents *in vitro* and *in vivo* [19]–[22].

Despite the increasing interest on the chemopreventive role of Se or green tea, it is unknown whether the combination of Se and green tea has a beneficial chemopreventive effect on CRC. Although preclinical and clinical reports of combining Se and green tea are lacking, combination approaches with Se or green tea have been studied in colon cancer and other cancer models [23]. For instance, a combination of Se and genistein has been shown to inhibit breast cancer in a rat model [24]; a combination of Se and vitamin E has provided greater protection against esophageal carcinogenesis in rats [22]. The combination of green tea and curcumin or the combination of green tea and sulindac has resulted in synergistic chemopreventive effect in an AOM CRC model [25], [26].

Se and green tea are particularly interesting as a combination not only because they can be easily co-administered in the diet, but also because they have potentially complementary mechanisms of action. Apoptotic removal and DNA repair enzyme O^6^-alkylguanine DNA alkyltransferase (MGMT)-mediated DNA repair are two important innate cellular responses to environmental carcinogen-induced oncogenic DNA lesions. Green tea has been shown to up-regulate MGMT activity in rat colon in our earlier study (unpublished data) by an epigenetic mechanism that would be expected to repair the type of adduct induced by azoxymethane (AOM) [27]. Dietary Se has been shown by our team to activate apoptotic deletion of AOM-affected cells [28]. We hypothesise that the risk of developing CRC will be reduced by combining agents that target different aspects of innate cellular responses to oncogenic DNA lesions. This is justified by the fact that CRC has a long initial latency period, involves multiple steps and pathways, and a combinational approach may simultaneously regulate multiple molecular and cellular targets involved in the process of CRC [29].

Our increased understanding of CRC at the epigenetic/genetic levels also opens up opportunities to interrupt and reverse the initiation and progression of CRC and provides many targets for dietary intervention [30]. The AOM-induced CRC model has been extensively used in both mechanistic and chemepreventive studies [31] because it recapitulates many of the clinical, pathological, and molecular features of human CRC, such as preneoplastic lesions, aberrant crypt foci (ACF), mutations in *K-ras* oncogene, and deregulation of signalling pathways in WNT/ß-catenin and inflammation [31], [32]. Although the roles of WNT-antagonists (such as secreted frizzled related proteins (SFRPs)), DNA methyltransferases (DNMT) and histone deacetylation in human colonic carcinogenesis are well documented, the expression of DNMT1, SFRR5 and acetylation of histone H3 in this model are largely unknown; which play crucial roles in the development and progression of human colon cancers [33]–[35]. The present study was designed to evaluate the chemopreventive effect of combining dietary agents of Se and green tea against colonic carcinogenesis, using ACF and colon tumors as endpoints. The effects of this combination on genetic/epigenetic biomarkers were also examined.

## Methods

### Reagents

AOM and green tea extract were purchased from Sigma-Aldrich Pty. Ltd. Green tea extract (P1204) contains 65% catechin including 34.5% Epigallocatechin Gallate (EGCG). Milk protein (0.34ppm Se) and Se-enriched milk protein (5ppm Se) were provided by Tatura Milk Industries (VIC, Australia) [28]. Se-enriched milk protein contains 83% selenomethionine, 5% selenocysteine and 4% unknown components [36].

### Animals

A total of 160 male Sprague-Dawley rats were obtained from the Animal Resource Centre, Adelaide University, Australia. The study was approved by the Animal Welfare Committee at Flinders University (# 651/07). Two experiments were performed, 60 rats for an ACF experiment and 100 for a long-term tumor experiment.

For each experiment, rats were divided randomly into 4 equal experimental groups (with comparable initial body weights), housed in plastic cages (four per cage) and maintained in a temperature and humidity-controlled animal facility with a 12 h light/dark cycle at 22±2°C temperature and 80±10% humidity. Rats were given free access to water, weighed weekly and were monitored closely for clinical signs of ill health throughout the study. Rats appearing sick were euthanized immediately.

### Diets

The experimental diets were based on a modified AIN-76A diet and contained 19% sunflower oil by weight so as to “humanize” the fat contribution to energy intake to ∼35% [28]. Milk protein was used as the protein source for control and green tea diets; Se-enriched milk protein was used as protein as well as a supplemental Se source for the high Se diet and the combination diet; control diet contained neither Se nor green tea. Choice of 0.5% green tea was based on our earlier study that this intake significantly increased MGMT expression (mRNA and activity) in rat colons (unpublished data). This dose contains 172.5 mg EGCG/100 g diet and provide 25.9 mg EGCG/rat/day, which when calculated on a per body weight basis would provide the equivalent intake in an adult human of 3–4 cups of green tea per day. This feeding regimen was well tolerated by animals [37]. 1ppm Se was chosen because our previous studies showed that Se-enriched milk protein at 1ppm significantly protected against colon cancers in mice [28]. The diets were prepared fresh at 4-weekly intervals, pelleted and stored at −20°C until used. Details of diets are provided in Table 1.

**Table 1:** Composition of experimental diets (g/100 g diet).

Ingredient	Control	Green tea	Se	Se + green tea
Milk protein#	20	20		
Se-enriched milk protein #,†			20	20
Sucrose	20	20	20	20
Corn starch	31.15	30.65	31.15	30.65
Fiber (alpha cell)	5	5	5	5
Sunflower oil	19	19	19	19
Choline	0.2	0.2	0.2	0.2
Mineral mix‡	3.5	3.5	3.5	3.5
Vitamin mix	1	1	1	1
Methionine	0.15	0.15	0.15	0.15
Green tea	0	0.5	0	0.5

The experimental diets consisted of a modified AIN-76A diet achieved by adding 19% sunflower oil and 20% protein.

# Milk protein was used as source of protein for control diet and green tea diet

† Se-enriched milk protein was used as source of protein and Se for Se diet and the combination diet.

‡ No Se was included in mineral mix in the diets.

### Experiment 1 (ACF study)

**Figure 1:**
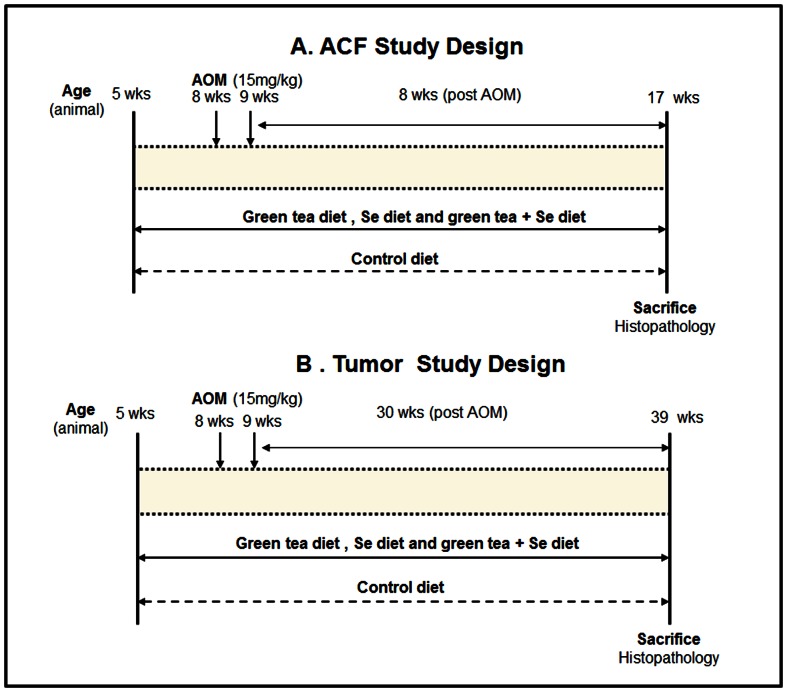
Experimental design for evaluation of Se, green tea and the combination diet for chemopreventive effects against colonic carcinogenesis in an AOM-induced rat CRC model. ACF study (A): groups of rats (n = 15) were fed control, Se, green tea or diet containing Se and green at age of 5 wk. 2 wk later, rats were given 15 (mg/Kg/body weight) AOM, once a week for 2 wk. 8 wk after last AOM treatment, rats were euthanized and colons were evaluated for ACF; normal-appearing crypts were also examined for β-catenin, COX-2 and Ki-67 expression. Tumor study (B) : groups of rats (n = 25) were fed control, Se, green tea or diet containing Se and green at age of 5 wk. Rats were given two weekly AOM treatments similar to ACF study. 30 wk after AOM treatment, rats were euthanized and colons were historically evaluated for tumor outcomes; normal-appearing crypts were also examined for β-catenin, DNMT1, Ac-H3 as well as *SFRP5* and *Cyclin D1* expression.

Beginning at 5 wk of age, rats (15/group) were fed to each of four diets. After 2 wk on diets, rats received 2 AOM injections (15 mg/Kg body weight) one wk apart. Rats remained on the same diet throughout the study until killed by CO_2_ asphyxiation 8 wk after the last AOM injection (Figure 1A). Colons were opened flat overnight on hibond C paper for ACF analysis. After analysis, a distal segment of normal-appearing colon (2 cm) was cut and stained with Ki-67, COX-2 and β-catenin antibodies (12/group).

### Experiment 2 (tumor study)

5 wk old rats (25/group) were fed to each of four diets, similar to ACF study. Rats received 2 weekly AOM injections (15 mg/kg) but were killed 30 wk after the last AOM (Figure 1B). Colons were examined for tumor endpoints and further processed for histopathologic evaluation. A distal segment of normal-appearing colon (2 cm) free of neoplasms was dissected for immunohistochemistry of β-catenin, DNMT1 and acetylated histone H3 (Ac-H3) (12/group) or frozen in liquid nitrogen for western blot analysis (6/group) or placed in RNAlater (Ambion) for quantitative RT-PCR analysis (6/group).

### Quantification of ACF

Colons were stained with 0.1% methylene blue solution and evaluated at ×40 magnification using a dissecting microscope in a blind scoring procedure. Total number of ACF in the entire colon was scored from the distal to the proximal end of the colon. ACF were distinguished from the surrounding normal crypts by their increased size, elevated appearance and the slit like shape of the luminal opening. Crypt multiplicity was defined as the number of crypts in each focus, and categorized as small (1–3 crypts/focus) and large ACF (4 or more crypts/focus).

### Histopathology of tumors

The number, location and size of each tumor were scored by an independent observer unaware of the dietary treatment. The tumors were categorized as adenomas and adenocarcinomas as described previously by us [28]. Endpoints were colon tumor incidence (i.e. proportion of rats with tumors, with adenomas or adenocarcinomas), tumor multiplicity (number of tumors/rat) and tumour size (tumor size/rat). Tumor size was calculated by the formula: log_10_ [∑(π(diameter1 + diameter 2))^2^/2] [38].

### Immunohistochemical analysis

The primary antibodies against Ki-67 (MIB-5, #M7248) were purchased from Dako, Australia; COX-2 (M-19-R, #SC-1747-R), β-catenin (E5, #SC-7963) and DNMT1 (H-300 #SC-2701) from Santa Cruz Biotechnology, Australia and Ac-H3 (Lys9/Lys14, #9677) from Cell signalling, USA. The detailed procedures for immunohistochemical analysis were reported previously [28]. In brief, antigen retrieval was carried out by heating sections in 0.1 M citrate buffer in pressure cooker plastic tub for 1 hour. Sections were incubated with Ki-67 antibody (1∶1000), COX-2 antibody (1∶500), β-catenin antibody (1∶1,000), DNMT1 antibody (1∶2,000), and Ac-H3 antibody (1∶5,000) overnight after incubation in 3% H_2_O_2_ for 20 mins. Detection for Ki-67 and COX-2 was biotinylated secondary rabbit-anti-mouse antibody (1∶200) (Dako) for 30 mins and avidin/biotinylated peroxidase complex (Signet Laboratories) for 20 mins. Detection for DNMT1 and β-catenin was by a HRP polymer link. Slides were visualized by incubating with 3′-diaminobenzamine substrate and counterstained with hematoxylin. A positive staining was identified by a reddish brown precipitate in the nucleus for Ki-67, DNMT1 and Ac-H3, in the cytoplasm for COX-2 and in membrane/nucleus for β-catenin. 20 intact perpendicular well-oriented normal-appearing crypts (extending from the muscular mucosa to the colonic lumen) were examined for each colon sample. The index of colonic crypt cells expressing Ki-67, COX-2, DNMT1 and Ac-H3 was calculated as the number of positive cells per crypt column divided by the total number of cells and multiplied by 100. Membrane and nuclear stained β-catenin cells were counted separately. The abnormal expression of β-catenin, DNMT1 and Ac-H3 was also examined in tumor tissues.

### Western Blot analysis

Aliquots of normal-appearing colon tissues from six rats per group were pooled and homogenized in ice-cold lysis buffer (50 mM Tris, 1% NP40, 0.5% sodium deoxycholate, 0.1% SDS, 5 mM EDTA, 2 mM PMSF and protease inhibitors) and centrifuged (14,000×g for 25 min at 4°C). The concentration of protein in supernatants was determined using the Bio-Rad protein assay. Equal amounts of proteins (30 µg) were separated on 4–20% SDS-PAGE gels, proteins were transferred to a nitrocellulose membrane using semi-dry transfer and the membrane was blocked with 5% skim milk, probed with β-catenin, DNMT1 and Ac-H3 antibodies and stained with secondary antibody (a horseradish peroxidase-labelled anti-rabbit IgG or goat anti-mouse IgG). Western blot was repeated three times for each sample. Immunoreactive proteins were detected using the enhanced chemiluminescence light detecting kit. Each membrane was re-probed with anti-β-actin antibody (Sigma) or anti-histone H3 antibody (#4499, Cell signalling). Band intensities for β-catenin, DNMT1were quantified by Image J and normalized with β-actin, whereas band intensity of Ac-H3 was normalized with H3 histone. Results are expressed as ratio to β-actin or H3 histone.

### Quantitative RT-PCR

Total RNA was extracted from rat colons using a QIAGEN RNeasy Mini Kit (Qiagen, Germany). The concentration and purity of the total RNA was estimated using a NanoDrop® ND-1000 UV-Vis spectrophotometer by measuring the absorbance at 260 and 280 nm. Complementary DNA (cDNA) was synthesized from 0.3 µg total RNA for each sample using a QIAGEN QuantiTect Reverse Transcription Kit. The *SFRP5* and *Cyclin D1* genes were co-amplified with *GAPDH* gene, which served as a housekeeping gene. Primers for *SFRP5* gene were purchased from Qiagne, Rn_Sfrp5_1_SG QuantiTect Primer Assays (NM_001107591, XM_001055342, XM_219887, XM_347305- Cat # QT01624056). Primers for *Cyclin D1*gene (NM_171992) were 5′-ATGAGAACAAGCAGATCATCC-3′ (Forward Primer) and 5′-TAGCAGGAGAGGAAGTTGTTG-3′ (Reverse Primer). Primers for the *GAPDH* gene (NM_017008) were 5′-AACATCATCCCTGCATCCAC-3′ (Forward Primer) and 5′-TTGAAGTCRCAGGAGACAAC-3′ (Reverse Primer). Real-time PCR was conducted with QuantiTect SYBR Green PCR Kit on a Rotor Gene 3000 Cycler (Corbett, Australia). The cycling conditions of 40 cycles were 94°C/15 s, 55°C/30 s and 72°C/30 s. Each sample was run in triplet repeat and normalized by *GAPDH*. For each PCR run, a non-template reaction was included as negative controls. All PCR data were analysed with Q-Gene software as we described previously [36], [39].

### Statistical analyses

Statistical analyses were performed using SPSS version 17.0 (SPSS Inc., Chicago, Illinois) and Stata version 11.1 (StataCorp, Texas). Results are expressed as means ± standard error of the mean for normally distributed data. Between-group comparisons for weight, Ki-67, COX-2, β-catenin, DNMT1, Ac-H3, *SFRP5* and *Cyclin D1* were performed using one-way ANOVA with correction for multiple comparisons by Tukey's post hoc test, a *P*-value of <0.05 was considered statistically between the groups. Between-group comparisons of ACF counts and tumor measures were assessed according to the 2×2 factorial designs with green tea and Se as the two main factors. Binary logistic and negative binomial regression models were employed to test for the main effects of green tea and Se and for possible interaction effects between green tea and Se. Post-hoc comparisons for each of the 3 intervention diets with the control diet as the comparison group, as well as comparison for the combination diet with the Se alone or green tea alone were also performed. A *P*-value of <0.05 was considered statistically significant for each main effect and interaction.

## Results

### General observation

Diets were well received and consumed by four rat groups. All rats regardless of dietary intervention group showed normal weight gain during the two experiments (data not shown); there was no significant difference in food consumption between the dietary groups. Examination of small intestine, liver and kidney did not reveal any abnormality in either study, indicating dietary supplementation with 1ppm Se or 0.5% green tea, or the combination of Se and green tea did not cause any overt toxicity. Six sick rats were killed before the termination of tumor study; they were distributed across the three treatment groups and tumor endpoints from these rats were not able to be included.

### The combination diet effectively inhibits ACF formation in AOM model

AOM-induced ACF were observed predominantly in the distal and middle colon. The effects of Se, green tea and the combination diet on ACF formation are shown in Table 2. Although the total number of ACF/rat from our study was somewhat lower compared to other previously published studies [40], rats fed green tea alone had no effect on total ACF and small ACF/rat, although it significantly reduced large ACF/rat, *P*<0.05, compared with those fed the control diet. In contrast, all categories of ACF crypt multiplicity (total, small and large ACF/rat) were significantly reduced by Se alone (*P*<0.05) and the combination diet (*P*<0.01), in comparison to the control diet. Importantly, the combination diet showed maximal inhibition on large ACF (i.e. ACF containing four or more crypt foci, the most relevant for predicting subsequent invasive tumor lesions) compared with either Se or green tea alone: with approximately 70% greater inhibition versus green tea diet and 66% inhibition greater versus Se diet (mean number  = 8.4±1.3, 7.5±1.6 and 2.5±0.5 for green tea, Se and the combination diet respectively, *P*<0.05 for the combination diet versus each of green tea and Se).

**Table 2:** Effect of Se alone, green tea alone, and their combination on the formation of AOM-induced ACF in rats.

Experimental diets	Green tea or Se in diet	Total ACF / per rat# (mean ± SEM)	Small ACF / per rat† (mean ± SEM)	Large ACF / per rat‡ (mean ± SEM)
Control diet	**0**	50.1±4.5^a^	37.3±2.6^a^	13.6±2.0^a^
Green tea diet	**0.5%**	42.4±7.2^a^	34.0±6.2^a^	8.4±1.3^b^
Se diet	**1 ppm**	30.7±4.3^b^	23.3±3.4^b^	7.5±1.6^b^
Se + green tea diet	**1 ppm + 0.5%**	27.3±5.6^b^	24.9±5.3^b^	2.5±0.5^c^

AOM, azoxymethane; Se, selenium; ACF, aberrant crypt foci; SEM, standard errors of mean.

ACF study was undertaken by feeding rats with 4 experimental diets (15 rats per group), containing either control diet, green tea diet, Se diet and the combination diet. Animals received two weekly AOM (15 mg/kg) injections to induce ACF and were killed 8 wk later.

# Total number of ACF was calculated as the sum of the small and large ACF.

† Small ACF (Crypt multiplicity) was classified by the number of crypts per focus (1–3).

‡ Large ACF (Crypt multiplicity) was classified by the number of crypts per focus (≥4).

Values with different superscripts (a, b, c) in each column were statistically different (*P*<0.05).

### The combination diet effectively inhibits tumor formation in AOM model

The significant decreases in the number of ACF are in keeping with the lower tumor burdens observed in the rats receiving the combination diet (Table 3). There was a significant interaction between Se and green tea, *P*<0.05, with the combination diet showing additive inhibitory effect on all tumor endpoints relative to a single dietary agent as green tea or Se, *P*<0.01. Tumor incidence, multiplicity and tumor size were reduced by 73.9, 80.4%, 73.4% versus green tea diet, and by 65.2%, 72.7%, 63% versus Se diet, respectively. Green tea alone did not significantly affect any tumor endpoint, while Se alone was less effective than the combination diet. Analysis of the effect of dietary intervention on cancer formation was specifically performed after histopathological examination of tumors. The incidence of adenoma in the control, green tea, Se and the combination diets were 26.1%, 20.8%, 16.7% and 4.3%, respectively. There was a significant reduction in the rats fed the combination diet, it was reduced by 79.3% versus green tea diet, and by 74.2% versus Se diet, *P*<0.01, respectively. The incidence of adenocarcinomas was reduced in a similar pattern; 17.4% in rats fed a control diet compared to 4.3% in those fed the combination diet, while in green tea alone the incidence was 12.5% and for Se alone the incidence was 8.3%. Notably, the combination diet reduced the incidence of adenocarcinomas by 65.6% versus green tea diet, and by 48.2% versus Se diet, but numbers were small and the results did not reach significance.

**Table 3:** Effect of Se and green tea alone, and their combination on tumor incidence, multiplicity and size in AOM-treated rats.

		Tumor incidence						Tumor multiplicity		Tumor size	
Experimental diet	No. rats	Rats with tumors (%)	% of inhibition	Rats with adenoma.(%)	% of inhibition	Rats with Adenocarci-noma#.(%)	% of inhibition	Tumors / rat (Mean ± SEM)	% of inhibition	Tumor / rat (Mean ± SEM)	% of inhibition
Control diet	23	10/23(43.5)^a^		6/23(26.1%)^a^		4/23(17.4%)		0.61±0.18^a^		0.80±0.20^a^	
Green tea diet	24	8/24(33.3)^a^	23.4%	5/24(20.8%)^a^	20.3%	3/24 (12.5%)	28.2%	0.46±0.15^a^	24.6%	0.64±0.19^a^	20%
Se diet	24	6/24 (25%)^b^	42.5%	4/24(16.7%)^a^	36%	2/24 (8.3%)	52.3%	0.33±0.11^b^	45.9%	0.46±0.17^b^	42.5%
Se + green tea diet	23	2/23 (8.7%)^c^	80%	1/23 (4.3%)^b^	83.5%	1/23 (4.3%)	75.3%	0.09±0.06^c^	85.2%	0.17±0.12^c^	78.7%

AOM, azoxymethane; Se, selenium; SEM, standard errors of mean.

Tumor study was undertaken by feeding animals with 4 different diets (25 rats per group), containing either control diet, green tea diet, Se diet and the combination diet. Animals received two weekly AOM (15 mg/kg) injections to induce tumor formation and killed 30 wk later.

# No statistical analysis undertaken for adenocarcinomas due to the small numbers.

Values with different superscripts (a, b, c) in each column were statistically different (*P*<0.05).

### The combination diet prevents AOM-induced ß-catenin nuclear translocation, COX-2 expression and cell proliferation

**Figure 2:**
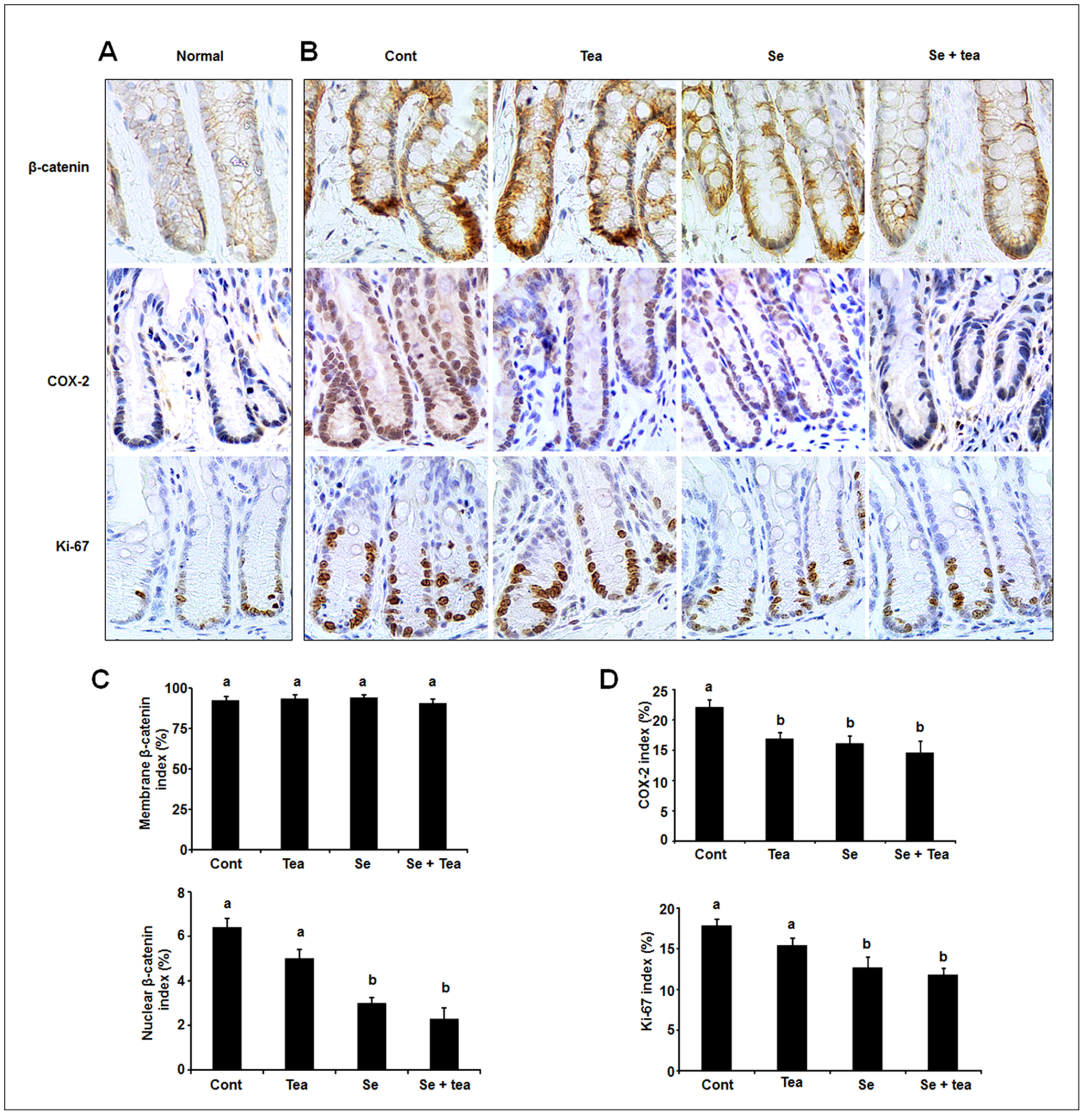
Effects of dietary Se, green tea and the combination of Se and green tea on β-catenin, COX-2 and Ki-67 expression (ACF study). A representative section of immunohistochemical staining of β-catenin, Ki-67and COX-2 in AOM-untreated normal crypts (A), AOM-treated normal-appearing crypts from control, green tea, Se and the combination diet (B); labelling index for β-catenin (membrane and nuclear positive cells, calculated as the number of positive cells per crypt column divided by the total number of cells and multiplied by 100 (n = 12) (C); labelling index for COX-2 and Ki-67 (n = 12) (D). β-catenin and Ki-67 nuclear staining was significantly decreased by the combination diet and Se alone, but not green tea. COX-2 cytoplasmic staining was significantly decreased by green tea, Se and the combination diet. Statistical significance of dietary intervention between the groups was analysed by ANOVA, values with different superscripts in each column were statistically different (*P*<0.05), Bars: mean ± SEM.

Effects of Se, green tea and the combination diet on ß-catenin, COX-2 and Ki-67 expression were examined in histologically normal-appearing crypts from the ACF study. We first compared the staining pattern of ß-catenin between AOM-untreated normal crypts and AOM-treated crypts. ß-catenin staining in normal crypts of untreated rats showed a low level of ß-catenin staining restricted to cell membrane, with limited nuclear staining (Figure 2A), whereas crypts from AOM-treated rats showed increased staining of ß-catenin in the nucleus (Figure 2B). Comparison of ß-catenin membrane and nuclear staining cells for the 4 dietary groups is shown in Figure 2B and Figure 2C. Se alone, green tea alone and the combination did not affect the percentage of cells showing positive ß-catenin membrane staining. But Se alone (*P*<0.05) and the combination diet (*P*<0.01) significantly reduced the percentage of cells showing positive ß-catenin nuclear staining, compared with the control, with no significant difference between Se alone and the combination diet, whereas green tea alone did not reduce ß-catenin nuclear staining. The percentage of cells showing Ki-67 nuclear staining was reduced in a similar pattern by Se alone (*P*<0.05) and the combination diet (*P*<0.01), green tea alone did not affect cell proliferation (Figure 2B and 2D), with a low level of Ki-67 staining in AOM-untreated normal crypts (Figure 2A). We also compared the staining pattern of COX-2 between AOM-untreated normal crypts and AOM-treated normal-appearing crypts. AOM untreated rats showed a low level of cytoplasmic staining for COX-2 in the colon (Figure 2A), whereas AOM-treated rats showed increased staining of COX-2 (Figure 2B). We found that the all three diets significantly inhibited COX-2 cytoplasmic staining (*P*<0.05), compared with control, there was no significant difference between the experiment diets (Figure 2B and 2D).

**Figure 3:**
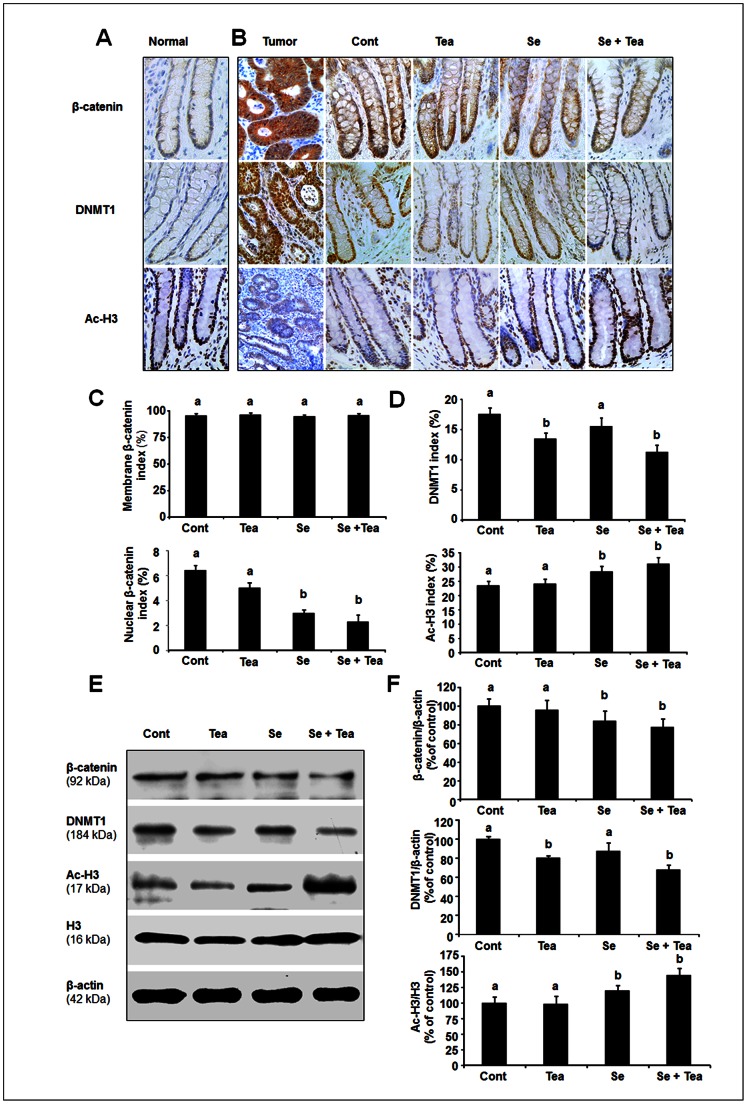
Effects of dietary Se, green tea and the combination of Se and green tea on β-catenin, DNMT1 and Ac-H3 expression (tumor study). A representative section of immunohistochemical staining β-catenin, DNMT1 and Ac-H3 in AOM-untreated normal crypts (A), AOM-treated normal-appearing crypts from the control, green tea, Se and the combination diet (B) and tumors; labelling index for β-catenin (membrane and nuclear positive cells) (n = 12) (C) and labelling index for DNMT1 and Ac-H3 (n = 12) (D). Western blot analysis of β-catenin, DNMT1 and Ac-H3 for colon samples (n  = 6) (E), expression of β-catenin and DNMT1 was normalized to that of β-actin and expression of Ac-H3 was normalized to that of H3, data was presented as precent of control (F). Increased strong β-catenin nuclear staining and DNMT1 overexpression were displayed in AOM-induced tumors, whereas weaker Ac-H3 expression was noted in colon tumors. β-catenin nuclear staining was significantly decreased by the combination diet and Se alone, but not green tea; DNMT1 was significantly decreased by the combination diet and green tea alone, but not Se; Ac-H3 nuclear staining was significantly increased by the combination diet and Se alone, but not green tea. Statistical significance of dietary intervention between the groups was analysed by ANOVA, values with different superscripts in each column were statistically different (*P*<0.05), Bars: mean ± SEM.

We next compared the staining pattern for β-catenin between AOM-treated normal-appearing crypts and AOM-induced tumors. Increased ß-catenin staining, in particular stronger nuclear staining was significantly higher in colon tumors than in normal-appearing crypts consistent with translocation of ß-catenin (Figure 3B). None of the diets significantly affected membrane staining for β-catenin in normal-appearing crypts from the tumor study, but Se diet alone (*P<0.05*) and the combination diet (*P<0.01*) showed consistent inhibitory effects on β-catenin nuclear accumulation (Figure 3C). The effect of dietary intervention on β-catenin expression was further supported by western blot analysis, showing total β-catenin was significant lower in normal-appearing crypts of rats fed the combination diet or Se alone (Figure 3E and 3F).

### The combination diet inhibits DNMT1 expression and increased histone H3 acetylation

DNA methyltransferases DNMT and histone modifications play important roles in DNA methylation, with overexpression of DNMT1 and deacetylation of histone H3 or H4 reported in colon cancer [33]. With this in mind we examined the staining pattern of DNMT1 and Ac-H3 in AOM-induced colon tumors, compared with AOM-untreated normal crypts as well as AOM-treated normal-appearing crypts. Strong nuclear staining of DNMT1 and less intense nuclear staining of Ac-H3 were noted in AOM-induced tumors (Figure 3B); relative to normal crypt (Figure 3A) and normal-appearing crypts (Figure 3B), where DNMT1 and AC-H3 positive cells were predominantly located in proliferative compartment (Figure 3B).

To determine whether DNMT1 and Ac-H3 in normal-appearing crypts were regulated by Se alone, green tea alone and the combination diets, we measured the percentage of DNMT1 and Ac-H3 positive cells for the 4 dietary groups (Figure 3D). Significant inhibition of DNMT1 was observed in rats fed the combination diet and green tea alone, *P*<0.05 versus control, whereas Se alone had no significant effect on DNMT1 expression. When comparing Ac-H3 between the dietary groups, strongest induction of Ac-H3 was noted in rats fed the combination diet, the Se alone also significantly increased Ac-H3, *P*<0.05, compared with control diet, but green tea alone had no effect (Figure 3D). The effect of dietary intervention on DNMT1 and Ac-H3 expression was also supported by western blot analysis, showing DNMT was significant lower in normal-appearing crypts of rats fed the combination diet or green tea alone, whereas Ac-H3 was significant higher in normal-appearing crypts of rats fed the combination diet or Se alone (Figure 3E and 3F). Our data also showed that changes of Ac-H3 levels were not reflected in total H3 protein because the H3 histone levels were not changed by dietary treatment.

### The combination diet restores *SFRP5* expression and inhibited *CyclinD1* expression

**Figure 4:**
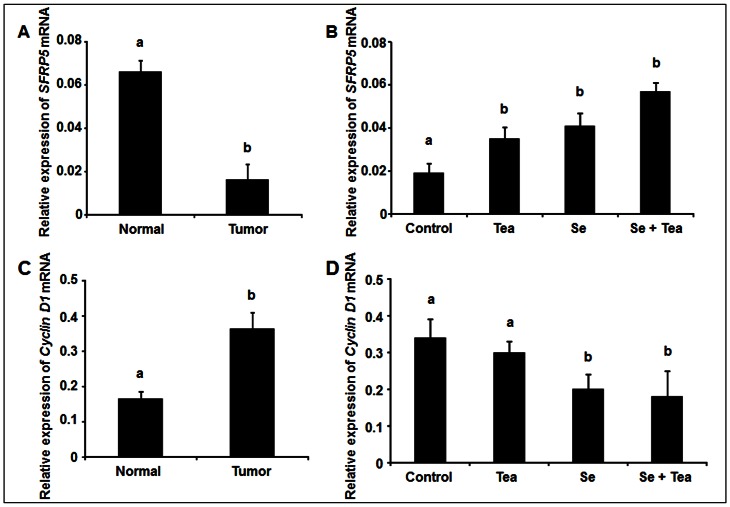
Effects of dietary Se, green tea and the combination of Se and green tea on *SFRP5* and *Cyclin D1* mRNA expression (tumor study). Quantitative RT-PCR analysis of expression of *SFRP5* (A) and *Cyclin D1* (C) mRNA in AOM-untreated normal colons and AOM-induced tumors (n = 6); expression of *SFRP5* (B) and *Cyclin D1* (D) mRNA from the control, green tea, Se and the combination diet (n = 6) in AOM-treated normal-appearing crypts. Significant repression of *SFRP5* and marked increase *Cyclin D1* mRNA in AOM-induced colon tumors were noted compared with normal colons. *SFRP5* was significantly increased by the combination diet, Se alone and green tea alone. *Cyclin D1* was also significantly inhibited by the combination diet and Se alone, but not green tea. Statistical significance of dietary intervention between the groups was analysed by ANOVA, values with different superscripts in each column were statistically different (*P*<0.05), Bars: mean ± SEM.

The WNT/β-catenin signalling pathway is regulated by several WNT antagonists, including SFRPs. *SFRP1, 2, 5* genes are frequently silenced by promoter hypermethylation in human CRC [41], [42], a high percentage of DNA methylation in *SFRP5* gene promoter has been detected by us in AOM-induced colon tumors (unpublished data). It is therefore important to compare the expression level of *SFRP5* and the oncogenic WNT/β-catenin downstream gene *Cyclin D1* between AOM-untreated normal colons and AOM-induced tumors. We found that levels of *SFRP5* mRNA were significantly lower in AOM-induced tumors, compared with AOM-untreated normal colons (Figure 4A), *P*<0.01, whereas the levels of *Cyclin D1* mRNA were markedly higher in AOM-induced tumors, relative to AOM-untreated normal colons (Figure 4C), *P*<0.05. These data suggest the activation of the WNT/β-catenin signaling pathway in response to AOM treatment. We next examined if dietary intervention regulates the gene expression of *SFRP5* and *Cyclin D1* in AOM-treated normal appearing-colons. Significant increased expression of *SFRP5* mRNA was observed in rats fed Se alone, green tea alone and the combination diet, compared to those fed control diet (Figure 4B), *P*<0.05. Notably, the level of *SFRP5* mRNA in response to the combination diet returned to the level similar to that of AOM-untreated normal colons. With regards to *Cyclin D1* expression, Se alone and the combination diet significantly inhibited *Cyclin D1* mRNA expression (Figure 4D), versus control, *P*<0.05, but green tea had no effect on *Cyclin* D1expression.

## Discussion

Apoptotic removal and MGMT-mediated DNA repair are two important innate cellular responses to environmental carcinogen-induced oncogenic DNA lesions. Our earlier studies in the AOM model provided evidence that Se-enriched milk protein at 1ppm prevents colon cancer by activating the acute apoptotic response [28]; and 0.5% green tea enhances MGMT expression (unpublished data) in rat colon. We have now extended these findings by showing that the combination of Se and green tea provides a significant protective advantage against the development of early preneoplastic lesions-ACF and colon tumors in the AOM model. The magnitude of inhibition of ACF (in particular large ACF) was consistent with the ability of the combination diet in inhibition of colon tumors (adenomas and adenocarcinomas), supporting the role of ACF as precursors to CRC. Se alone also significantly inhibited most of tumor endpoints, but to a degree less than that found with the combination diet, whereas green tea alone had no significant effect on colonic oncogenesis. Our results are of considerable applied significance because the combination of dietary and/or chemopreventive agents with different modes of actions is emerging as an attractive strategy for chemoprevention [2], [43].

The relative low potency of dietary agents compared with pharmacological compounds makes combination of two distinct dietary agents particularly attractive for achieving greater efficacy for cancer prevention [20]. The challenge is to identify an effective combination, with dietary agents working through similar or different mechanisms to produce an additive or synergistic chemopreventive effect. Se and green tea work through complementary mechanisms targeting different innate cellular responses to oncogenic DNA lesions and so might be valuable. While this is the first study to examine the beneficial effect of a combination of Se and green tea in an animal model of CRC, the concept of combination of Se and green tea is not new. Se-enriched tea powder had a higher antimutagenic effect than Se or tea alone in mice bone marrow, suggesting a possible synergistic interaction between Se and tea ingredients [44]. Supplementation of Se-enriched tea extracts also yielded better inhibitory effects than regular tea on transplanted hepatocellular carcinoma in mice [45]. Thus additional preclinical animal studies of combination of Se and green tea are important before translating this combination regimen to human dietary intervention trials.

Given the challenge for people to drink large amounts of green tea due to its bitterness, a “pill”-based approach to providing green tea is likely to be necessary. Green tea leaves could be ground into micronized powders and added into food to increase the absorption of nutrients in green tea. Oral administration in food appears to be a better route of administration than drinking as this delivered a two-fold increase in EGCG to the small intestine [46]. The choice of Se-enriched milk protein over inorganic Se compounds was simply due to this source being readily incorporable into a range of food products. While the dose of 1ppm Se-enriched milk protein used in the present study was several fold greater than that used for human intervention studies (200 µg Se/day) [5], our previous studies indicated that 1ppm Se was at the lowest range of Se forms to be effective in the AOM model, Se-enriched milk protein at 0.5ppm also showed a trend towards protection [28]. The cancer-protective effects of other Se forms in animals, however, occurred at a level of intake about ten-fold greater than those found to be effective in human [10]. Therefore, it is plausible that supplementation of Se-enriched milk protein at a relatively low doses together with green tea may also be of benefit for CRC prevention.

We further examined the molecular mechanisms that may underlie the combination effects of Se and green tea. Nuclear ß-catenin translocation is the hallmark of activation of the WNT signalling pathway [47] and overexpression of inflammatory markers of COX-2 and increased cell proliferation have been shown in the AOM CRC model from early focal lesions through to adenocarcinomas [48]. Previous studies have suggested that the effects of AOM and dietary agents on biomarkers (such as Cyclin D1and iNOS) are reflected by parallel changes in the ACF and histologically normal-appearing crypts, suggesting the involvement of similar mechanisms between ACF and normal-appearing crypts [49], [50]. In this regard, we examined the effect of the combination diet on ß-catenin, COX-2 and cell proliferation in normal-appearing crypts. In agreement with previous findings [51], increased ß-catenin nuclear translocation was accompanied by increased COX-2 and cell proliferation, which were significantly inhibited by the combination diet in both ACF and tumor studies. Moreover, the chemopreventive effect of Se, green tea and their combination were closely related to the degree of inhibition of ß-catenin and cell proliferation, supporting the view that the WNT signalling pathway is critical for the regulation of colonic crypt renewal and homeostasis [52] and β-catenin is an important biomarker for CRC chemoprevention. Given that the ß-catenin mediated signalling pathway has been identified as a key regulator of epithelial proliferative response [53], the chemopreventive effect of the combination diet might be mediated through a cascade of events in which the primary inhibitory effect may be on ß-catenin, followed by cell proliferation.

Targeting DNA methylation and histone modifications with dietary compounds has emerged as a promising chemopreventive strategy in human clinical trials [54]. Essential micronutrients such as folate, vitamin B-12, Se as well as the dietary tea and sulforaphane are among a growing list of agents that affect epigenetic events as novel mechanisms of chemoprevention [33], [55]. Since Se and green tea have been reported to have DNMT and histone deacetylase (HDAC) inhibition activity in *in vitro* [56], this prompted us to test the hypothesis that Se and green tea might affect DNMT1 and histone acetylation and thereby inhibit colonic tumorigenesis. The findings of overexpression of DNMT1 and reduced Ac-H3 in AOM-induced tumors suggest the clinical relevance of this model for CRC research. More importantly, we found that although Se alone did not significantly inhibit DNMT1 expression, the expression of DNMT1 was significantly inhibited by the combination diet, green tea alone also markedly inhibited DNMT1. When comparing the effects of dietary intervention on increased expression of Ac-H3, significant effects were achieved only with the combination diet or with Se alone. It has been reported that naturally occurring organoselenium compounds can act as novel HDAC inhibitors because they share structural features with butyrate (a short-chain fatty acid) reported to competitively inhibit HDAC activity [56]. The important finding from the present study is that Se-enriched milk protein might act as an HDAC inhibitor; it has yet to be determined whether a particular chemical form of Se in dairy protein may be responsible for its effect on histone acetylation. Nevertheless, induction of histone acetylation appears to be a potential mechanism for Se in cancer prevention. While green tea may also act as a DNMT inhibitor in AOM model, its inhibitory effect on oncogenesis is not apparent.

The findings that Se and green tea may act as an HDAC or DNMT inhibitor are very interesting. It extends our current understanding of how combination of Se and green tea may prevent CRC by enhancing innate cellular responses to oncogenic lesions, and provides a premise for targeting DNMT1 and histone acetylation for CRC prevention. It is reasonable to propose that the inhibition of DNMT and HDAC in response to combination of green tea and Se may lead to up-regulation of tumor suppression gene or down-regulate proto-oncogenes. This would advance our understanding by postulating that green tea and Se, on top of their already understood actions, bring the epigenetic pathway into play. WNT antagonist gene *SFRP5* is a member of SFRPs family and is frequently down-regulated in human CRC [34], [35]. We examined the effect of the combination diet on the expression of *SFRP5*, and a WNT downstream target gene *Cyclin* D1 in AOM-treated normal-appearing colons because hypermethylation of promoter *SFRP5* (unpublished data), epigenetic silencing of *SFRP5* gene and activation of the WNT pathway (accumulation of β-catenin nuclear translocation and increased *Cyclin D1* gene expression) were observed by us in AOM model. We found that the combination diet resulted in restoration of *SFRP5* gene expression to the levels similar to that of normal colons, which were accompanied by significant changes in the expression of a number of WNT-related gene/proteins in a pattern consistent with inhibition of WNT activation, such as inhibited β-catenin nuclear accumulation, reduced *Cylin D1* expression and cell proliferation. These results suggest that epigenetic mechanisms may be involved in activation of the WNT signalling pathway for colon cancer development in AOM model and potential dietary manipulation can be developed using WNT pathway genes/proteins as useful biomarkers for CRC prevention. It has been shown that genistein attenuates the WNT signaling by up-regulating *SFRP2* in a human colon cancer cells through changes in demethylation of CpG islands of *SFRP2* promoter region [57], and the anthocyanins derived from black raspberries demethylates tumor suppressor genes of *SFRP2, 5* and *WIF1* through inhibition of DNMT1 and DNMT3B [58]. In a human study, up-regulation of three WNT-antagonist genes by blackberries was associated with decreased DNMT expression [42].

In conclusion, this study showed that a combination of dietary Se and green tea more effectively prevents ACF and colon tumor formation than either agent alone. The preventive effect is associated with regulating genetic and epigenetic biomarkers implicated in colonic carcinogenesis, as evidenced by restoring *SFRP5* gene expression, increasing histone H3 acetylation and reducing DNMT1 expression, inhibiting ß-catenin nuclear accumulation, reducing *Cyclin D1* expression and cell proliferation in normal-appearing crypts. This combination is thus promising for use in the chemoprevention of CRC but before this can be recommended with confidence, more preclinical studies and clinical trials are needed to validate this novel combination regimen.
